# MALARIA—WHY DO MOSTLY CHILDREN GET SICK?

**DOI:** 10.3389/frym.2024.1305938

**Published:** 2024-02-01

**Authors:** Rolando Garza, Mischa Huson, Anakaren Garcia, Bella Gonzalez, Kenneth Musinguzi, Avani Nagaragere, Evelyn Nansubuga, Maato Zedi, Evelien M. Bunnik, Sebastiaan Bol

**Affiliations:** 1 Department of Microbiology, Immunology and Molecular Genetics, University of Texas Health Science Center, San Antonio, TX, United States; 2 Department of Internal Medicine, Radboud University Medical Center, Nijmegen, Netherlands; 3 South Texas Undergraduate Research Opportunities Program (STUROP), University of Texas Health Science Center, San Antonio, TX, United States; 4 Voelcker Biomedical Research Academy (VBRA), University of Texas Health Science Center, San Antonio, TX, United States; 5 Infectious Diseases Research Collaboration, Tororo, Uganda

## Abstract

Did you know that micro-organisms can live in blood? *Plasmodium* parasites can infect red blood cells and cause a serious disease called malaria. This disease is mostly seen in young children living in Africa. Sick children have a fever, aches, can feel very tired, and in bad cases, they can even die from malaria. There are medicines that cure malaria, but it is hard to get these to everyone who needs them. Fortunately, as children grow older, they do not feel as sick when they are infected by the malaria-causing parasite. Better yet, adults hardly ever get malaria. The reason for this difference between children and adults has to do with how well the body’s defense system can fight off the parasite. Keep reading if you want to learn more about malaria, the *Plasmodium* parasite and how the immune system fights against it.

## WHAT IS MALARIA?

**Malaria** is a disease caused by a germ or **micro-organism** that can live in blood. This micro-organism is a **parasite** named *Plasmodium*. Humans can get infected with this parasite when they are bitten by a mosquito that carries the *Plasmodium* parasite. The word “malaria” comes from the Italian words “mala aria”, which means “bad air”. A long time ago, people thought that breathing in the bad air coming from smelly swamps caused this disease. Later, in the late 1800s, scientists learned that it is not something bad in the air that causes malaria, but instead malaria is caused by a parasite [1] that is transmitted from mosquitoes to humans [2, 3]. The standing water of swamps was simply a perfect place for mosquitoes to live. This explains why malaria was seen in areas with standing water.

Parasites are small, living things that need to live on or inside other organisms—called hosts—for food. Parasites are unwanted guests that can make their host very sick. The *Plasmodium* parasite has two hosts—it needs to live inside mosquitoes *and* humans. The life cycle of the *Plasmodium* parasite is complex because it involves two hosts, and different shapes (life stages) of the parasite.

## HOW DOES THE PARASITE GROW INSIDE HUMANS?

The *Plasmodium* parasite needs to live inside the human body to survive. But where exactly in the body does the parasite live and multiply? When a mosquito “bites” a human, it actually drinks a little bit of blood. Female mosquitoes need blood to make eggs. When the snout of an infected mosquito is in the skin of a human, *Plasmodium* parasites can enter the human body. These parasites first travel to the liver, one of the biggest organs, located just below the heart. The parasites grow in the liver for about a week and undergo an important change: they become able to infect red blood cells, which is bad news! Red blood cells carry oxygen from the lungs to all parts of the body.

After a *Plasmodium* parasite has entered or infected a red blood cell, it starts to eat the cell’s hemoglobin—the molecule that binds oxygen. Even worse, inside the red blood cell, the parasite grows bigger and multiplies. After about 2 days, there are so many new parasites inside the red blood cell that it bursts open, releasing the parasites and dying in the process. At some point there may not be enough red blood cells left to carry oxygen to the tissues. Furthermore, infected red blood cells can become sticky and block the body’s blood vessels [4]. Blocked blood flow can prevent important organs, like the brain, from receiving enough oxygen.

**Figure 1:**
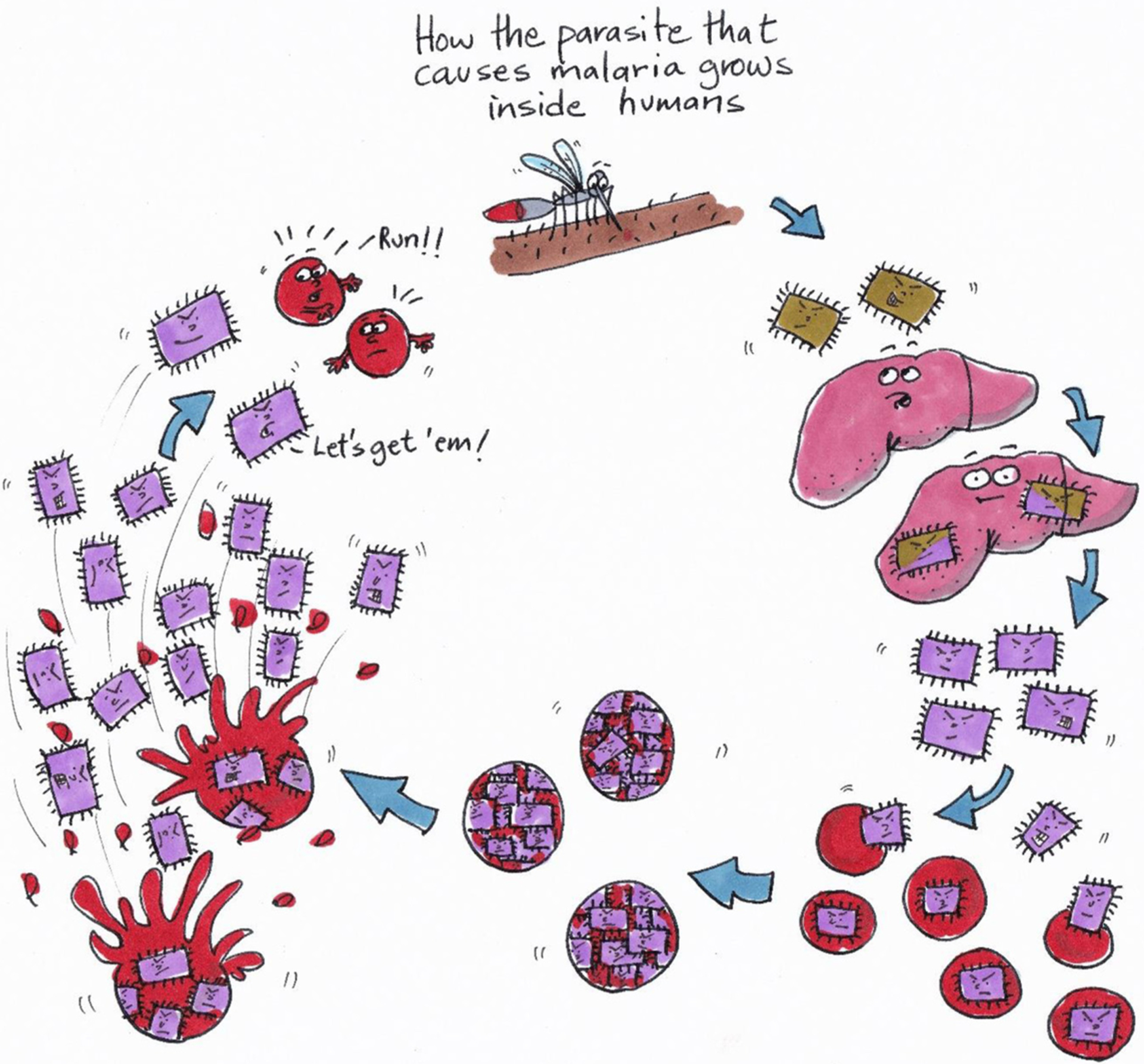
When an infected mosquito bites a human, the parasites (brown squares) move into a blood vessel and travel to the liver (top right). Here, the parasites multiply, and many new parasites leave the liver (purple squares). The parasites can now infect red blood cells (bottom right). Once inside a red blood cell, the parasite divides many times and, after about 2 days, dozens of new parasites burst out of a single red blood cell (left). When this happens, the red blood cell gets destroyed. Every new parasite can now infect a new red blood cell again. Bad news for the red blood cells!

With infected red blood cells flowing through the blood vessels, the next time a mosquito “bites” an infected human, the mosquito can become infected, too. Now, this newly infected mosquito can bite and infect another person. This completes the circle of the *Plasmodium* parasite life cycle (Figure 1).

## HOW DOES IT FEEL TO HAVE MALARIA?

Most people with malaria have a fever and feel very weak. With a fever, the body is too hot—sometimes malaria fevers can even reach 40°C (104°F), and people sweat a lot. Other symptoms are headaches, chills, aching muscles, feeling extremely tired, and not wanting to eat. When people are very sick, their urine can be the brownish color of cola, which is caused by the presence of destroyed red blood cells in the urine. These symptoms result from the *Plasmodium* parasites living and multiplying in the red blood cells.

Malaria is a serious disease and people with malaria need medicines quickly to make them better. If they are not treated within a couple of days, it is possible that not enough oxygen will reach their important organs, including the brain. When this happens, people can go into a coma, meaning they become unconscious and cannot wake up. Most malaria patients in a coma will die if they do not receive medicines.

## WHO GETS MALARIA?

**Figure 2:**
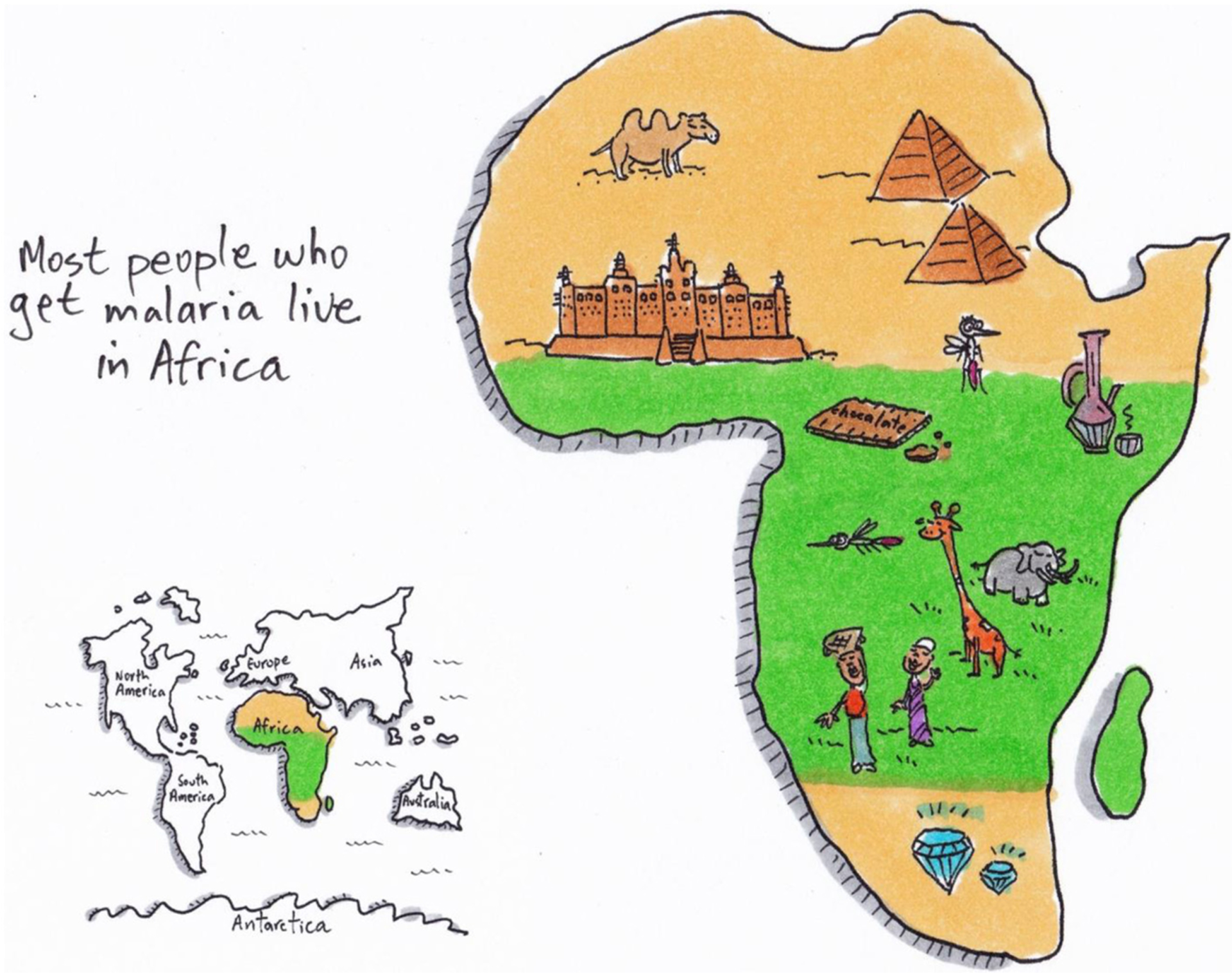
Africa is one of the world’s seven continents, and it has more than 50 countries. It is an extremely beautiful and diverse continent with amazing nature, animals, and culture. Most people in Africa live in the tropical (green) region. The tropics are a great place for mosquitoes to live: there is lots of water and it never gets too cold for the mosquitoes. This geographical factor is not the only reason most malaria cases are seen in Africa. In many African countries there is not enough money to protect people against the parasite and prevent malaria.

Malaria is a common disease that kills a lot of people—and some other animals as well. Millions of people get malaria and every minute a young child dies of malaria. Almost all (~95%) malaria patients live in Africa (Figure 2) [5]. Of all the malaria patients in the world who die of this disease, three out of four are young children [5]! Together with diarrhea, malaria is the most common cause of child death in Africa. Compared to the USA or Europe, 15 times more young children die in Africa.

Unlike any of the other six continents, most of Africa lies in the tropics—areas of the world around the equator where it is warm and wet. This is the climate mosquitoes like best. There are many different types of mosquitoes. The *Anopheles* mosquito that carries the most dangerous *Plasmodium* parasite, *Plasmodium falciparum*, prefers to bite humans more than other animals, and it likes to live in Africa. This is why there are many *Plasmodium* infections in Africa—most of them with the dangerous *Plasmodium falciparum* parasite. On top of that, in many African countries, there is not enough money available to prevent people from getting sick.

Anyone living in the tropical parts of Africa is at high risk of getting malaria, no matter what color their skin is. However, people visiting Africa, including tourists, often do not get malaria, because they have the opportunity to take medicines that will prevent them from getting sick when bitten by an infected mosquito. This type of prevention is called prophylaxis.

## WHY DO MOSTLY CHILDREN GET MALARIA?

As we mentioned, 75–80% of the people dying from malaria are young children. Why do so few adults get malaria, while there are many more grownups than children? Most adults who get infected with the *Plasmodium* parasite feel a little sick, but they do not need medicines to get better—they do not get malaria. Why is this?

**Figure 3:**
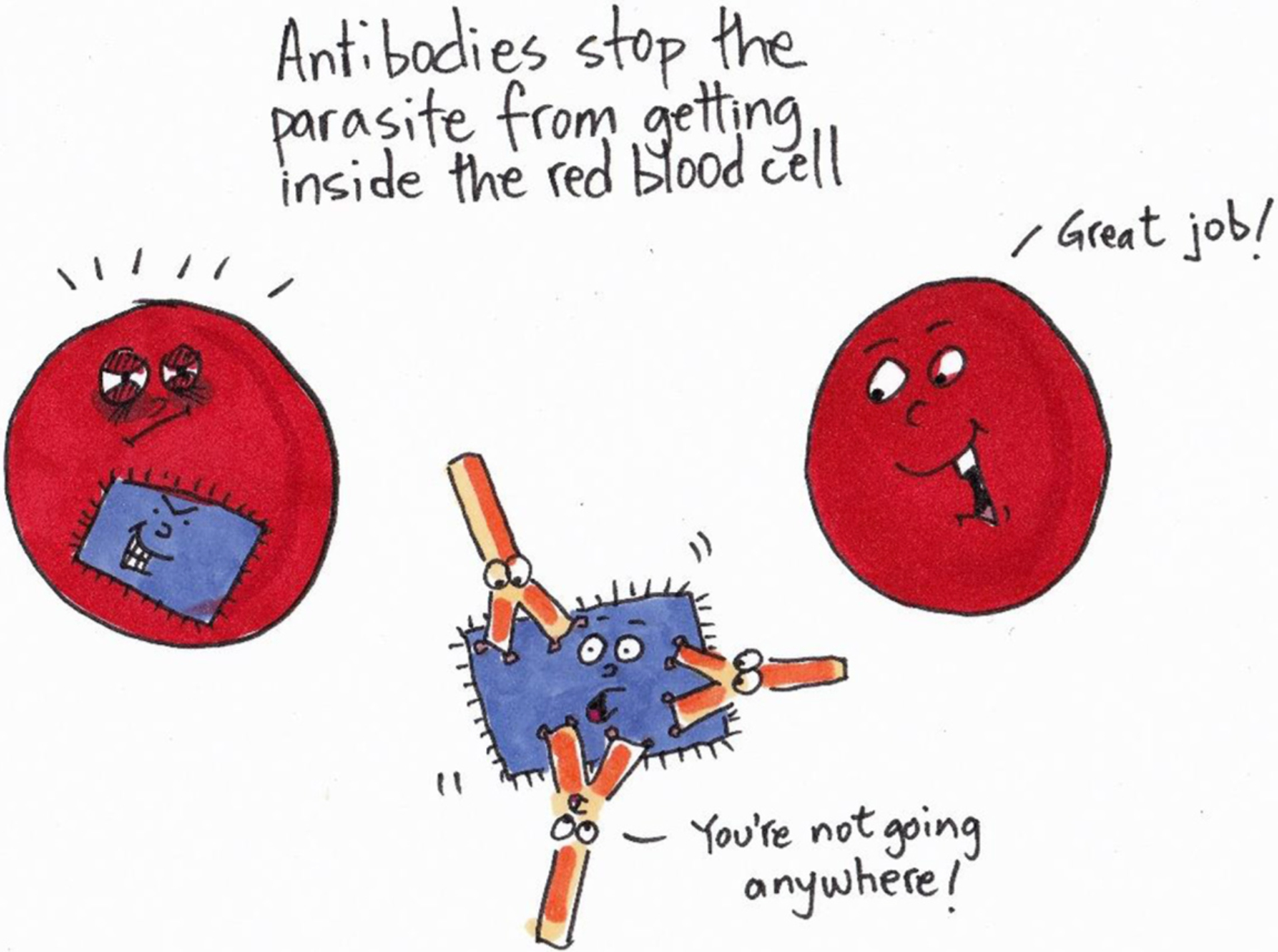
When antibodies (yellow/orange) bind to the parasite (blue), the parasite can no longer invade the red blood cell. When the parasites cannot get inside the red blood cells, the red blood cells stay alive, and the person does not get sick.

Most adults living in Africa have been infected with the *Plasmodium* parasite many times in their lives. When they were children, they probably got malaria several times. As they grew older, their bodies learned how to better deal with the parasite. The part of the body that fights parasites and other germs is called the **immune system**. This defense system consists of many different white blood cells, their products (for example **antibodies**), and several organs (for example bone marrow, spleen, and thymus). B cells—produced inside bone—are a small, special group of white blood cells that produce antibodies. Antibodies are proteins that stick to germs (for example *Plasmodium* parasites), thereby preventing them from entering a human cell (for example a red blood cell) and making the person sick (for example getting malaria) (Figure 3).

**Figure 4:**
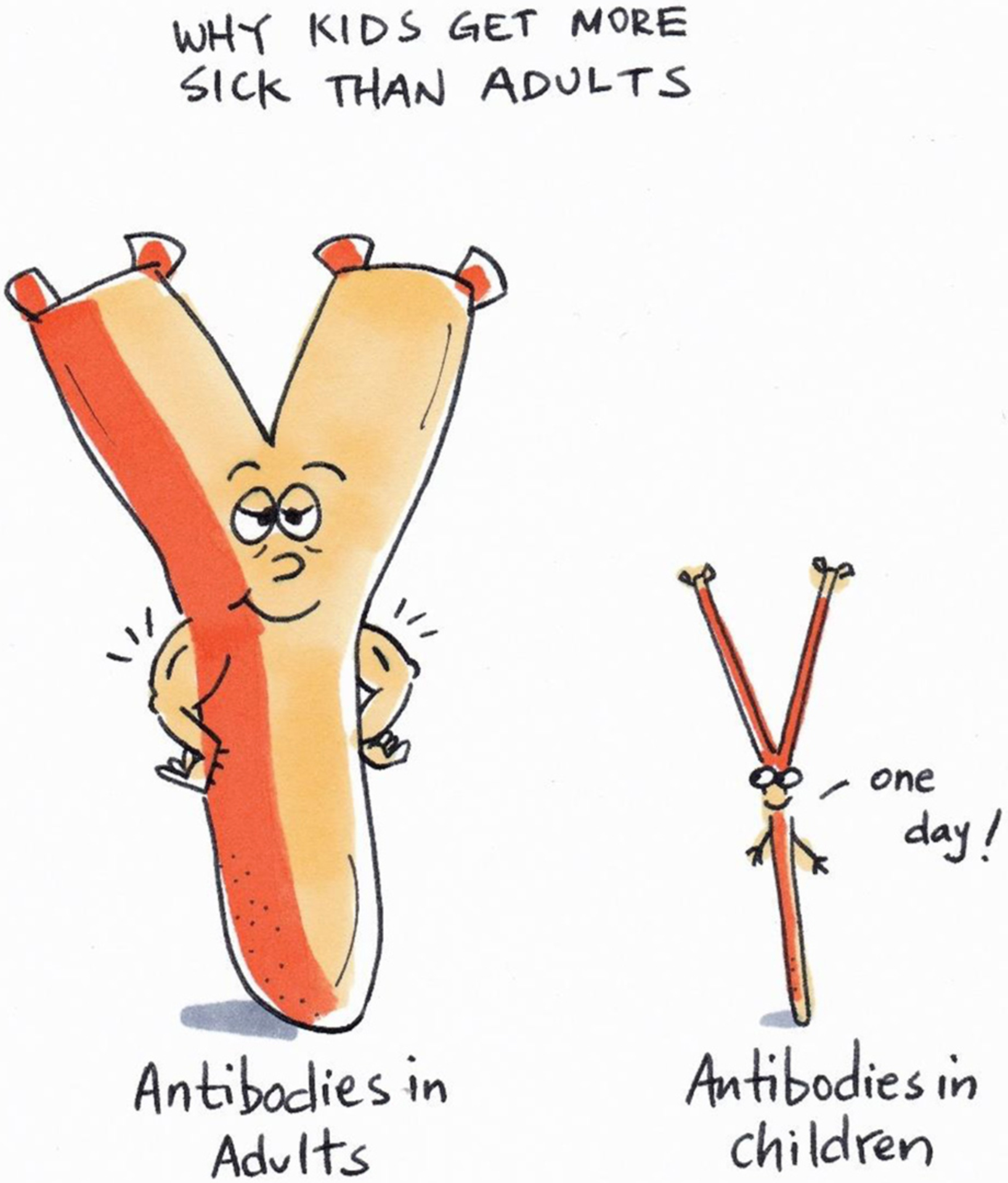
The immune or defense system of a child cannot yet make effective (big and strong) antibodies against *Plasmodium* parasites yet. A child’s immune system also does not make as many antibodies as adults do. Not having enough effective antibodies is believed to be the main reason why mostly children get malaria, while adults do not get as sick when they are infected by the parasite. It takes time and many infections for the immune system to grow up and be strong enough to fight off the parasite.

Antibodies also help in other ways than blocking the invasion of germs. They can stick to a germ and kill it, or get other cells of the immune system to eat it. An important study done in the early 1960s showed that antibodies found in the blood of grownups who had malaria many times, could be used to cure children with malaria [6]. However, making B cells that can produce strong and enough antibodies takes several years. Every time the immune system sees the malaria-causing parasite, it learns and becomes better at making more and stronger antibodies the next time it sees the same germ. This is probably why mostly young children get malaria—their antibodies are not good enough yet at defending the red blood cells against *Plasmodium* parasites (Figure 4).

## HOW CAN WE GET RID OF MALARIA?

The easiest way to prevent people from getting malaria is to prevent them from getting bitten by infected mosquitoes. Malaria has been around for thousands of years and also used to be a big problem in Europe and the USA. In the 19^th^ and 20^th^ centuries, European countries and the USA spent a lot of money getting rid of standing water and killing mosquitoes by spraying chemicals. All people with malaria were given anti-malaria medicines for free. Since the 1950s and 1970s, these places have been considered malaria free.

The number of people dying from malaria has decreased by 30% in the last 20 years [5], meaning that fewer people get malaria now than a long time ago. However, about 500,000 young children still die of malaria each year, and a lot still needs to be done before no one gets malaria anymore. Eliminating malaria may be possible if *all* countries of the world contribute and people all over the world work together. Different methods must be combined, such as building more hospitals, training more nurses and doctors, building houses with window screens, providing bed nets, making anti-malaria medicines available and *affordable* for everyone, and spraying chemicals against mosquitoes. At the same time, scientists must keep working on discovering new and better anti-malaria medicines, and a more effective vaccine. (For more information about vaccines and how they work, see this, this, or this Frontiers for Young Minds article).

## Abbreviations

MALARIA: A disease caused by the *Plasmodium* parasite, which is spread by mosquitoes. Malaria patients have a fever, and they can die when not treated. Most patients are children living in Africa.
MICRO-ORGANISM: A living thing so small that it can only be seen under a microscope. Micro means small. Examples of micro-organisms are bacteria, viruses and some small parasites.
PARASITE: A living thing that needs to live on or inside another living being—called the host—to survive. It feeds and multiplies in a way that hurts the host.
RED BLOOD CELLS: Cells in the blood that carry oxygen from the lungs to all tissues in the body. These cells color blood red.
IMMUNE SYSTEM: All cells, things these cells produce (for example antibodies), and tissues in the body that help protect an animal against disease-causing germs.
ANTIBODIES: Y-shaped proteins, made by a type of white blood cells called B cells, that can stop a micro-organism from making a person sick. B cells and antibodies are part of the immune system.
